# Harnessing endophytic fungi for biosynthesis of selenium nanoparticles and exploring their bioactivities

**DOI:** 10.1186/s13568-022-01408-8

**Published:** 2022-06-08

**Authors:** Heba G. Hussein, El-Sayed R. El-Sayed, Nahed A. Younis, Abd El Hamid A. Hamdy, Saadia M. Easa

**Affiliations:** 1 Plant Research Department, Nuclear Research Center, Egyptian Atomic Energy Authority, Cairo, Egypt; 2 Chemistry of Natural and Microbial Products Department, National Research Center, Giza, Egypt; 3 Microbiology Department, Faculty of Science, Ain Shams University, Cairo, Egypt

**Keywords:** Selenium nanoparticles, Antioxidant, Antifungal, Antibacterial, Endophytic Fungi, Mycosynthesis

## Abstract

In the light of the fast growing several applications of selenium nanoparticles (SeNPs) in different industrial and agricultural sectors, this paper was conducted to explore the suitability of endophytic fungi as nano-factories for SeNPs. Thus, 75 fungal isolates were recovered from plant tissues and tested for their efficacy to biosynthesize SeNPs. Four promising strains were found able to synthesis SeNPs with different characteristics and identified. These strains were *Aspergillus quadrilineatus* isolated from the twigs of *Ricinus communis*, *Aspergillus ochraceus* isolated from the leaves of *Ricinus communis*, *Aspergillus terreus* isolated from the twigs of *Azadirachta indica*, and *Fusarium equiseti* isolated from the twigs of *Hibiscus rose-sinensis*. The synthesized SeNPs were characterized by several techniques viz., UV–Vis, X-ray diffraction, Dynamic light scattering analyses, High resolution transmission electron microscopy, and Fourier transform infrared spectroscopy, to study their crystalline structure, particle sized distribution, and morphology. Furthermore, the *in vitro* antimicrobial and antioxidant activities were evaluated. SeNPs synthesized by the four strains showed potent antifungal and antibacterial potentials against different human and phyto- pathogens. Moreover, SeNPs synthesized by the respective strains showed promising antioxidant power with IC_50_ values of 198.32, 151.23, 100.31, and 91.52 µg mL^− 1^. To the best of our knowledge, this is the first study on the use of endophytic fungi for SeNPs’ biosynthesis. The presented research recommends the use of endophytic fungi as facile one-pot production bio-factories of SeNPs with promising characteristics.

## Key points

- Discovery of four different promising endophytic fungi for a facile-synthesis of SeNPs.
- SeNPs were successfully mycosynthesized and characterized.
- SeNPs exhibited promising antifungal, antibacterial, and antioxidant activities.

## Introduction

Selenium (Se), a trace mineral that has an indispensable role in a biological system. It is a fundamental element in the diet, needed for health and growth of human, animals, and microorganisms. As such, their vital and important roles in the formation of many seleno- proteins and enzymes (Fardsadegh and Jafarizadeh-Malmiri 2019). Recently, selenium nanoparticles (SeNPs) have gained a considerable attention in the fields of food science and biomedicine due to the higher biocompatibility and lower toxicity than their counterparts; elemental Se, selenite and selenate (Tran et al. 2015; Wadhwani et al. 2016). Recent studies reported the wide range of biological and therapeutic activities of SeNPs such as antimicrobial (El-Sayed et al. 2020a), antiprotozoal (Shirsat et al. 2015), anticancer (Huang et al. 2013; Wadhwani et al. 2016), free radicals scavenging (El-Sayed et al. 2020a), and DNA protection against oxidative damage (Battin et al. 2011). Along with the aforementioned activities, SeNPs attracted great attention of researchers in the field of photo-conducting, (Srivastava and Mukhopadhyay 2013), optics and electronics (Barnaby et al. 2011), and catalytic degradation of pollutants (Zhang and Spallholz 2011). Accordingly, they are highly acceptable and recommended as promising candidates for several medical, industrial, and agricultural applications.

SeNPs are currently synthesized by several physical and chemical techniques (Gunti et al. 2019). However, these technologies are of high economic costs and extremely complicated. Besides, their negative impact on the environment through the resultant hazardous toxic wastes, which limit their use in biological applications as well (Shirsat et al. 2015). Hence, there is a pressing necessity to develop a cost-effective and cleaner production of SeNPs. Generally, the search for new microbial strains and screening their capability for synthesis of nanomaterials have emerged as a fast-growing and interesting research area for exploring possible future improvements of the process of nano-biosynthesis (Grasso et al. 2021). Certainly, microbial as bio-factories of these NPs are highly recommended in the last decade due to their promising capabilities for large-scale applications (El-Sayed et al. 2020b), modification (Dorcheh and Vahabi 2016), and improvement (Mousa et al. 2021).

Despite that, some microbes have been used in the preparation of SeNPs (El-Sayed et al. 2020a), only few fungal species have been reported as SeNPs-synthesizers. In general, fungi-mediated synthesis of several nanomaterials have magnificently emerged as the most advantageous (Grasso et al. 2021). Over the years, fungi have been employed extensively in several industrial applications (Dorcheh and Vahabi 2016; El-Sayed and Zaki 2022) as the most efficient biotechnological factories due to their flexibility, simplicity, easy maintenance, and tolerance. In this regard, the use of endophytes has emerged as a new research area for green and cost-effective production of several NPs. This novel research area will open a new horizon, on the interface of nanotechnology and microbiology, for novel synthesis of diverse nanomaterials with several applications (Abdelhakim et al. 2020; El-Sayed et al. 2022a). Today, these endophytes started to find their application in different nanotechnology fields because their metabolic activity is much higher than their free counterparts (Mousa et al. 2021; El-Sayed et al. 2022b). For all of these reasons, we aim in this paper to explore the effectiveness of fungal endophytes of some plant species as facile, environmentally friendly and cost-effective bio-factories of SeNPs. As the first step in this direction, 75 fungal species were recovered from plant tissues and tested for their ability to synthesize SeNPs. The facile one-pot synthesis and characterization of SeNPs using four fungal strains were described for the first time. Furthermore, the antimicrobial and antioxidant potentials of SeNPs synthesized by the four strains were explored.

## Materials and methods

### Plant samples and isolation of fungal endophytes

Samples were removed by a sterile sharp blade from healthy plant parts. Table 1 presents the used plant species. Plant species were identified from different cultivated locations in Egypt. The collected plant samples were transported to the laboratory and used for isolation of fungal endophytes according to El-Sayed et al. (2020c). Samples were fragmented to small parts and surface sterilized with 70% ethanol (for 1 min) followed by 0.1% HgCl_2_ (for 1 min), washed with sterile dist. water, and let to dry on a sterile filter paper. All parts were transferred aseptically to on the surface potato-dextrose (amended with streptomycin and tetracycline) agar plates and were incubated at 25 °C, then checked daily. The isolated cultures were checked for purity and stored in glycerol (15%) as a suspension of spores and mycelia at − 4 °C (El-Sayed et al. 2020d).

**Table 1:** Fungal endophytic genera isolated from different plant species and their ability to reduce sodium selenite salt

Host plant		Fungal genera								
		*Alteranria*	*Acremonium*	*Aspergillus*	*Penicillium*	*Fusarium*	*Mucor*	*Botrytis*	*Trichoderma*	*Cladosporium*
*Ricinus communis*	T	1	–	1*	1	–	–	–	–	–
	L	1	–	1*	–	–	–	1	1	–
*Malus domestica*	T	–	1	–	–	1	–	–	–	–
	L	1	–	1	1	–	–	1	1	–
*Azadirachta indica*	T	–	–	1*	–	–	–	–	1	1
	L	1	–	–	1	1	–	–	–	–
*Cupressus sempervirens*	T	–	1	–	1	–	–	–	–	–
	L	1	1	1	–	–	–	–	1	–
*Hibiscus rose–sinensis*	T	1	–	–	–	1*	1	1	–	–
	L	–	–	1	–	–	–	–	–	–
*Cinnamomum camphora*	T	–	1	–	1	–	–	1	–	–
	L	–	–	1	–	1	–	–	1	–
*Citrus medica*	T	–	–	–	–	–	–	–	–	1
	L	1	1	1	–	1	–	1	–	–
*Psidium guajava*	T	–	–	–	–	–	1	–	–	–
	L	–	1	1	–	–	–	–	–	–
*Citrus sinensis*	T	–	–	–	–	–	–	–	1	–
	L	1	–	1	1	–	–	1	–	–
*Mangifera indica*	T	–	1	1	–	–	–	–	–	–
	L	–	–	–	–	1	–	–	–	–
*Rhamus aurea*	T	–	–	1	–	–	–	–	–	–
	L	1	1	–	–	–	1	–	–	–
*Pinus sylvestris*	T	1	–	1	–	–	–	–	1	–
	L	–	1	–	–	1	–	–	–	–
*Terminalia arjuna*	T	–	–	–	1	–	–	–	–	–
	L	1	1	1	–	–	–	–	–	–
	B	–	–	–	–	–	–	–	–	1
*Chorisia crispiflora*	T	–	–	–	–	–	–	–	–	1
	L	1	–	1	–	1	1	–	1	–
	B	–	–	–	–	–	–	–	–	1
Total		12	10	15	7	8	4	6	8	5

PDA was used for endophytic fungi isolation. *T* Twig, *L* Leaf and *B* Bark

Filter-sterilized sodium selenite solution (1 mM) was added to the PDA plates and incubated for 7 days at 30 ℃

*Indicates a red color change to the cultivation medium

### Screening the isolated endophytes for their biosynthetic ability of SeNPs

The ability of fungal isolates to synthesize SeNPs was first detected using solid medium then in broth medium. Spore suspension for each fungal isolate was harvested from 7-days old culture and the final spore concentration was adjusted using a hemocytometer to 10^6^ mL^− 1^. 1 mL of this spore suspension was added under aseptic conditions to the surface of PDA plates containing 1 mM filter-sterilized sodium selenite (Na_2_SeO_4_, Sigma Aldrich, MO, USA) solution. Simultaneously, negative control of un-inoculated PDA plates containing sodium selenite salt and positive control of inoculated PDA plates without selenite salt were maintained under the same conditions. All plates were incubated at 30 ℃ for 7 days. The visible change in the color of the fungal colony to red was considered a positive result for the bio-reduction of Na_2_SeO_4_ amended in the medium and thereby SeNPs synthesis.

In another set, 1 mL of the prepared spore suspension of each fungal strain was transferred to 250 mL Erlenmeyer flasks containing 50 mL potato-dextrose broth (PDB, with pH adjusted to 6.0) amended with 1 mM filter-sterilized sodium selenite. Simultaneously, negative control of un-inoculated PDB flasks containing sodium selenite salt and positive control of inoculated PDB flasks without selenite salt were maintained under the same conditions. All flasks were statically incubated at 30 °C for 7 days. The visible change in the color of the broth medium to red was considered a positive result for the bio-reduction of Na_2_SeO_4_ salt.

### Fungal strains

Several endophytic fungal isolates were screened for the synthesis of SeNPs (as described earlier). Among the isolated fungi four different isolates were able to reduce sodium selenite salt and used for the preparation of SeNPs (as described later). These strains were *Aspergillus quadrilineatus* isolated from the twigs of *Ricinus communis*, *Aspergillus ochraceus* isolated from the leaves of *Ricinus communis*, *Aspergillus terreus* isolated from the twigs of *Azadirachta indica*, and *Fusarium equiseti* isolated from the twigs of *Hibiscus rose-sinensis*. The four strains *A. terreus*, *A. quadrilineatus*, *F. equiseti*, and *A. ochraceus* were identified and deposited under numbers AUMC14392, AUMC14393, AUMC14394, and AUMC14395 in the Culture Collection (aun.edu.eg/aumc/aumc.htm) of Assiut University Mycological Center, Egypt.

### Identification of the positive endophytic fungi

Identification of the four strains was accomplished by colony morphology, growth characteristics, and molecular characterization. Morphological identification was performed by studying the colony morphology on Czapek’s-yeast autolysate (CYA) agar according to Moubasher (1993). CYA (g L^− 1^): yeast extract 5, sucrose 3, NaNO_3_ 3, KH_2_PO_4_ 0.5, MgSO_4_.7H_2_O 0.5, KCl 0.5, and FeSO_4_.7H_2_O 0.01. Furthermore, the appearance of hypha and spores was examined under microscope after staining with lactophenol cotton blue stain.

Molecular characterization was performed according to the method by White et al. (1990) using PCR-amplified ITS1-5.8 S-ITS2 rRNA-gene. In brief, DNA of the fungal strains were extracted and sequenced by Solgent Company, Daejeon, South Korea. The composition of the used primers were Fw ITS1 (5′-TCC GTA GGT GAA CCT GCG G-3′) and ITS4 (5′-TCC TCC GCT TAT TGA TAT GC-3′). Sequences of the four strains were submitted to the GenBank and accession numbers were received. Finally, sequences of the four strains were analyzed using the online tool BLAST (http://www.ncbi.nlm.nih.gov/) and the software BioEdit (version 7.0.1). A neighbor-joining tree with the maximum-likelihood for each fungal strain were constructed using MEGA software version 6.0.

### Separation, purification, and characterization of SeNPs

After 7 days of incubation, culture broths of the four positive fungal strains was used to separate the produced SeNPs. Cultures were filtered through Whatman No0.1 filter paper and SeNPs were collected by the centrifugation for 20 min at 20,000 rpm. The collected SeNPs were washed in ethanol thrice then in deionized water and dried in a hot air (50 °C) oven. Fine powders of SeNPs obtained from each fungal strain were separately dissolved in ethanol, prior to characterization, and treated ultrasonically for their dispersion (El-Sayed et al. 2020a).

SeNPs from each fungal strain was characterized by the following techniques; UV-Vis spectroscopy (3101PC Spectrophotometer, Shimadzu, Japan), X-ray diffraction (XRD) patterns recorded in the range 20°≤2ϴ≤80° (Cu-K*α* radiation, wavelength of 1.5406°A, at 40 KV, and 40 mA) using a D8 DISCOVER BRUKER diffractometer DAVINCI design, USA), Dynamic light scattering (DLS) analyses (Zetasizer Nano ZS, Malvern Instruments, Worcestershire, UK), High resolution Transmission Electron Microscopy (JOEL Transmission Electron Microscope, model 2100, Japan), and Fourier Transform Infrared spectroscopy recorded at 400–4000 cm^− 1^ (FT-IR, IRAffinity-1 spectrophotometer, Shimadzu, Japan).

### In vitro Antimicrobial sensitivity tests

SeNPs from each fungal strain were separately dissolved in methanol (HPLC-grade) at different ratios to obtain the following concentrations 31.250, 62.50, 125, 250, 500, and 1000 µg mL^− 1^. All the concentrations were treated ultrasonically. The antifungal and antibacterial potentials of SeNPs were investigated according to Pongtharangkul and Demirci (2004) by the agar-well diffusion assay. Wells (5 mm) were cut through the agar by a sterile cork porer. The antifungal assay was against two plant pathogens *Fusarium oxysporum* EUM37 and *Alternaria alternata* EUM108 (obtained from the Microbiological Resources Centre, Cairo MIRCEN, Faculty of Agriculture, Ain Shams University, Cairo, Egypt) and two human pathogens *Aspergillus brasiliensis* ATCC16404 and *Candida albicans* ATCC10231. Under the same conditions, a positive control of Nystatin/Fluconazole (standard antifungal) and methanol only were also applied. Meanwhile, the antibacterial was evaluated against different human pathogenic bacteria *Pseudomonas aeruginosa* ATCC15442, *Bacillus cereus* ATCC10876, *Staphylococcus aureus* ATCC6538, *Klebsiella pneumoniae* ATCC13883, *Bacillus subtilis* ATCC6633, and *Escherichia coli* ATCC11229. Under the same conditions a positive control of Amoxicillin/Azithromycin acid (standard antibiotic) was applied. After incubation, zones of inhibition were measured carefully and the MIC values (minimum inhibitory concentration) of SeNPs were recorded from wells containing the lowest concentration of SeNPs with an inhibition zone.

### In vitro Antioxidant activity

SeNPs from each strain were separately dissolved in HPLC-grade methanol to get the concentrations range of 25–1000 µg mL^− 1^ then treated ultrasonically. The antioxidant potential of SeNPs was estimated by the free radical scavenging assay using 2,2^\^-diphenyl picrylhydrazyl (DPPH), Sigma-Aldrich, St. Louis, MO, USA according to the method described by Thaipong et al. (2006). Simultaneously, a positive control of ascorbic acid (Sigma-Aldrich, St. Louis, MO, USA) at the same range of concentrations range was also tested. Scavenging activity (%) was calculated as the change in the absorbance of the mixture (DPPH + SeNPs) with respect to the DPPH solution only (control). Moreover, IC_50_-values were calculated for graphic plot of each SeNPs using the software GraphPad Prism, San Diego, USA.

### Statistics

The recorded results were expressed as the mean taken from triplicate measurements from three independent experiment ± the standard deviation. Significant differences were determined at 95% confidence intervals using the analysis of variance (ANOVA) test followed by Dunken’s test (SPSS software v. 22, IBM, NY).

## Results

### Isolation and screening of fungal endophytes

Fourteen different plant species were used to isolate endophytic fungi (Table 1) from different plant parts including twigs, leaves, and bark. A total of 75 fungal isolates were recovered from the plant samples on PD agar plates. The isolated endophytes were subjected to a preliminary morphological examination to detect their genera. The obtained results showed that the 75 isolates belong to nine genera as follows: *Cladosporium*, *Trichoderma*, *Botrytis*, *Mucor*, *Fusarium*, *Penicillium*, *Aspergillus*, *Acremonium*, *and Alteranria*.

**Fig. 1:**
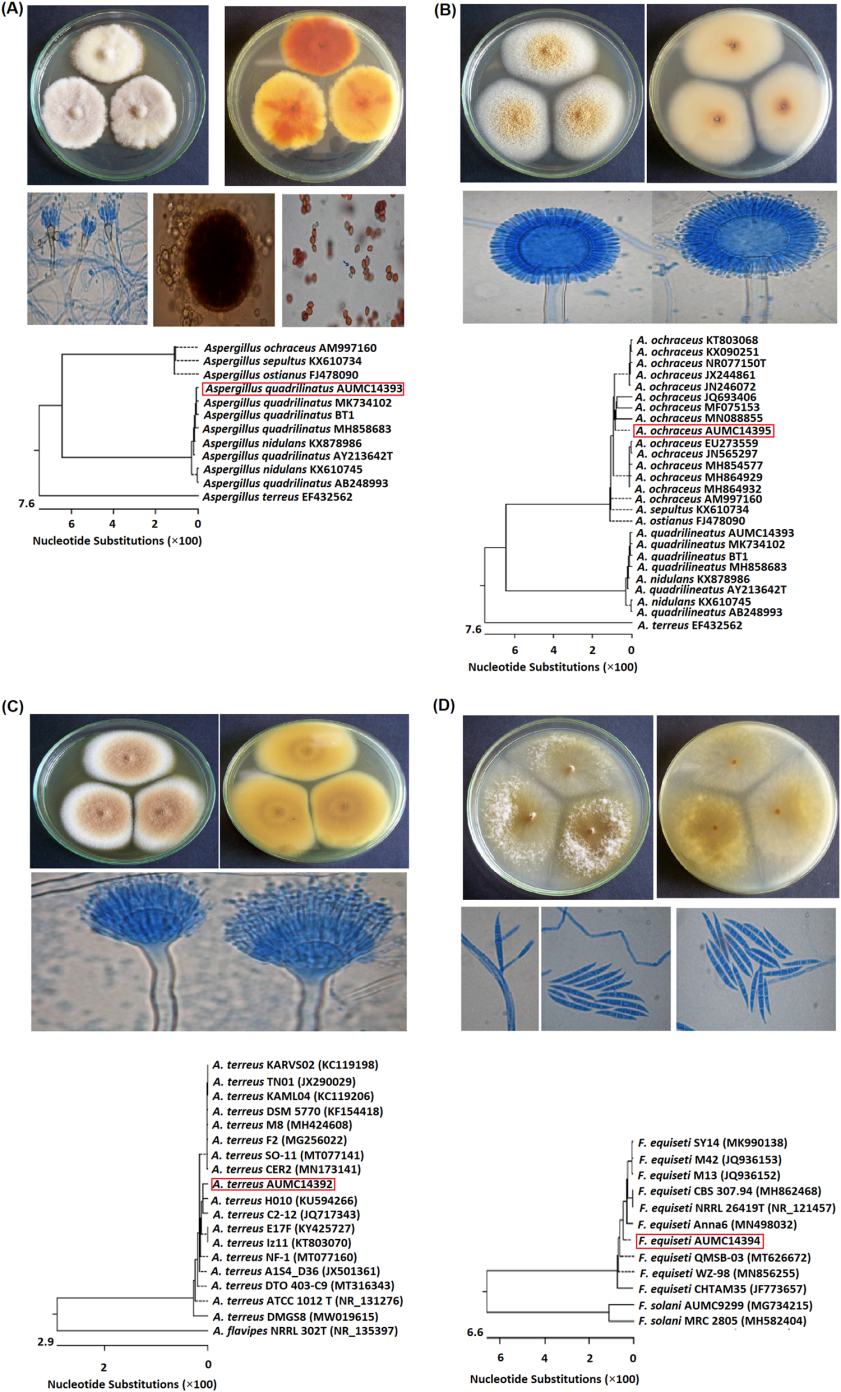
Morphological and molecular characteristics of the SeNPs producing fungal strains. Colonial growth, microscopic features, and phylogenetic tree of *A. quadrilineatus* MZ571533 (**A**), *A. ochraceus* MZ571534 (**B**), *A. terreus* MZ571560 (**C**), and *F. equiseti* MZ571561 (**D**)

The 75 isolates were separately inoculated on the top of PDA plates and PD broth amended with 1 mM sodium selenite to detect their ability to synthesize SeNPs. Table 1 clearly showed that of the 75 isolates only 4 isolates were positive. The four strains showed the ability to reduce selenite salt to selenium where a red color was developed, as indicated in Fig. 1A–D Insets. As a result, the four fungal strains among the 75 isolates were selected for further production and characterization of SeNPs.

### Morphological and molecular characterization

Figure 1A showed the colony morphology of *Aspergillus quadrilineatus* grown on CYA-agar plates. The strain had a broad growth with white-colored colonies with irregular margins in the right view at 25 °C after 10 days. Meanwhile, the reverse view showed an orange brownish pigmentation of the colony. Microscopic appearance showed conidiophores each with a columnar head bearing metulae, phialides and conidia. Hyaline Hulle cells surrounding the ascoma (cleistothecium) which contains asci and ascospores. Moreover, reddish purple ascospores each with 4 equatorial cristae (arrowed). Sequences obtained from molecular characterization were deposited in the GenBank under number MZ571533, then analyzed and a phylogenetic tree was developed. The obtained data (Fig. 1A) confirmed the high similarity of the fungus with *A. quadrilineatus* MK734102, MH858683, BT1, and AY213642T strains.

Figure 1B showed the colony morphology of *Aspergillus ochraceus* maintained on the CYA-agar. The fungus showed light ochraceous buff to golden yellow colonies (left) and buff brownish pigmentation of reverse (right) after 10 days of growth on CYA medium at 25 °C. Microscopic appearance showed conidiophores with radiate conidial heads (globose vesicles bearing metulae, phialides and chains of small conidia). Sequences were deposited in the GenBank under number MZ571534. Data presented in Fig. 1B indicated that the fungus had a high similarity with *A. ochraceus* KX090251, NR077150T, MN088855, and MF075153 strains.

Figure 1C showed the colony morphology of *Aspergillus terreus* maintained on the CYA-agar with a velvety, cinnamon to light brown colonies (left) and orange brownish pigmentation of reverse (right) after 10 days of growth on CYA medium at 25 °C. Microscopic appearance showed conidiophores with vesicles bearing metulae, phialides and chains of small conidia. Sequences were deposited in the GenBank under number MZ571560 then analyzed. Figure 1C confirmed the high similarity of the fungus with *A. terreus* MK734102, MH858683, BT1, and AY213642T strains.

Figure 1D showed the colony morphology of *Fusarium equiseti* maintained on the CYA-agar with floccose colonies, yellow to brownish aerial mycelium (left) and yellow brownish pigmentation in reverse (right) after 10 days at 25 °C of growth on CYA medium. Microscopic appearance showed hyphae, conidiogenous cell and septate, beaked conidia. Moreover, fusiform conidia each with 3–5 septations, beaked apex and foot-shaped basal cell were observed. Sequences were deposited in the GenBank under number MZ571561 then analyzed. Figure 1D showed confirmed the high similarity with *F. equiseti* NRRL26419T, MH862468, JQ936152, and JQ936152 strains.

### Biosynthesis and characterization of SeNPs

**Fig. 2:**
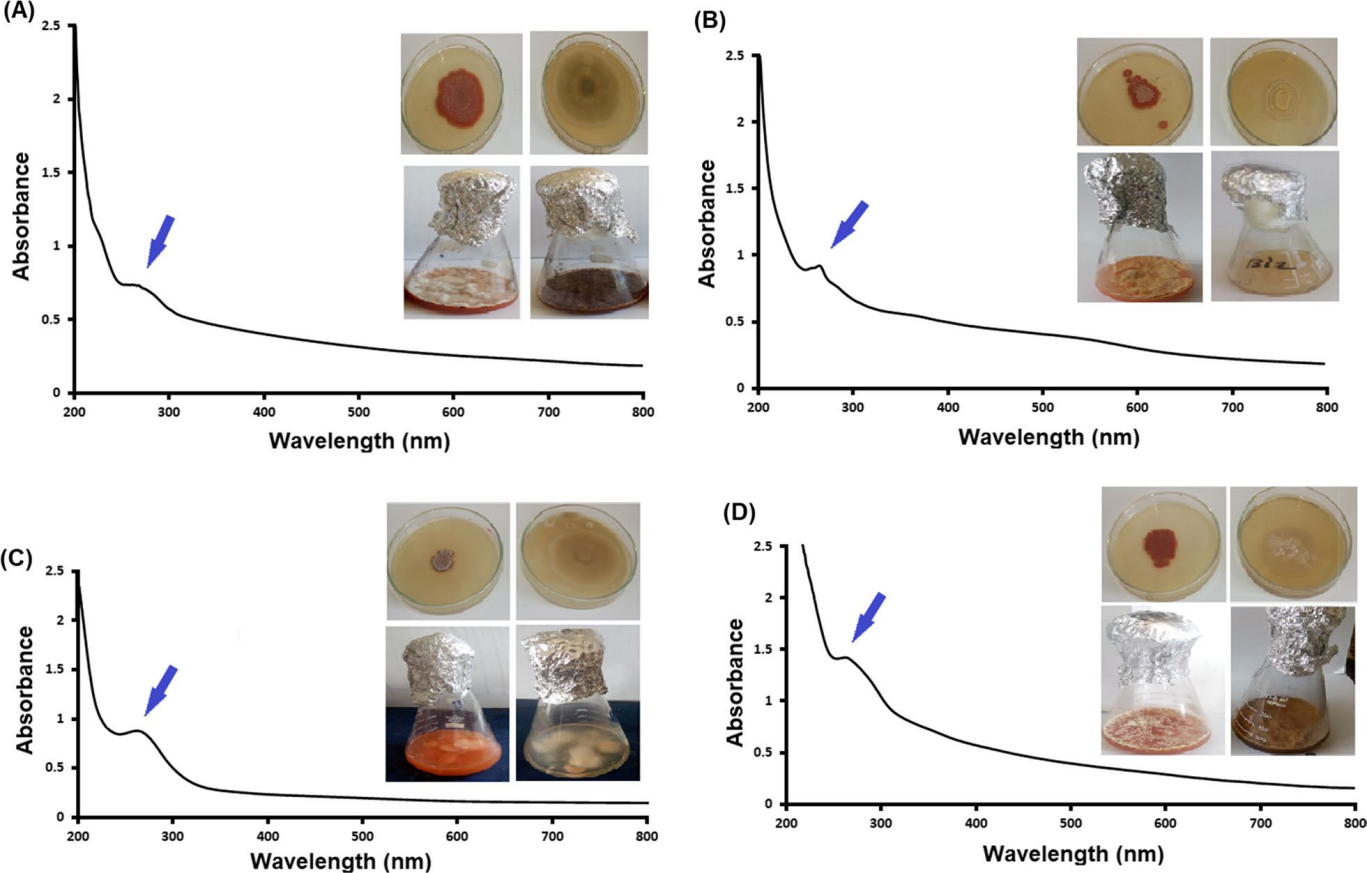
UV-Vis spectra of SeNPs. Fig. Inset shows PDA plates and PD broth flasks with (left-handed) and without sodium selenite (right-handed). NPs were separated from the culture broth, purified, dissolved in methanol and treated ultrasonically then the absorption was recorded in the range 200–800 nm. **A** UV-Vis spectrum of SeNPs synthesized by *A. quadrilineatus*, **B** UV-Vis spectrum of SeNPs synthesized by *A. ochraceus*, **C** UV-Vis spectrum of SeNPs synthesized by *A. terreus*, and **D** UV-Vis spectrum of SeNPs synthesized by *F. equiseti*

Sodium selenite was added to the cultivation medium of the endophytic fungal isolates separately. At the end of incubation period, the bio-reduction of the selenite slat to SeNPs was observed. The colorless broth media were turned to red color as shown in Fig. 2A–D-Insets.

#### UV–Vis spectroscopy

UV–Vis spectrophotometry of the powder from each fungal strain was analyzed to confirm the formation of SeNPs. In the recorded spectra of the four types of the synthesized SeNPs (Fig. 2A–D), the maximum absorption peak was observed at 265 nm.

#### XRD analysis

**Fig. 3:**
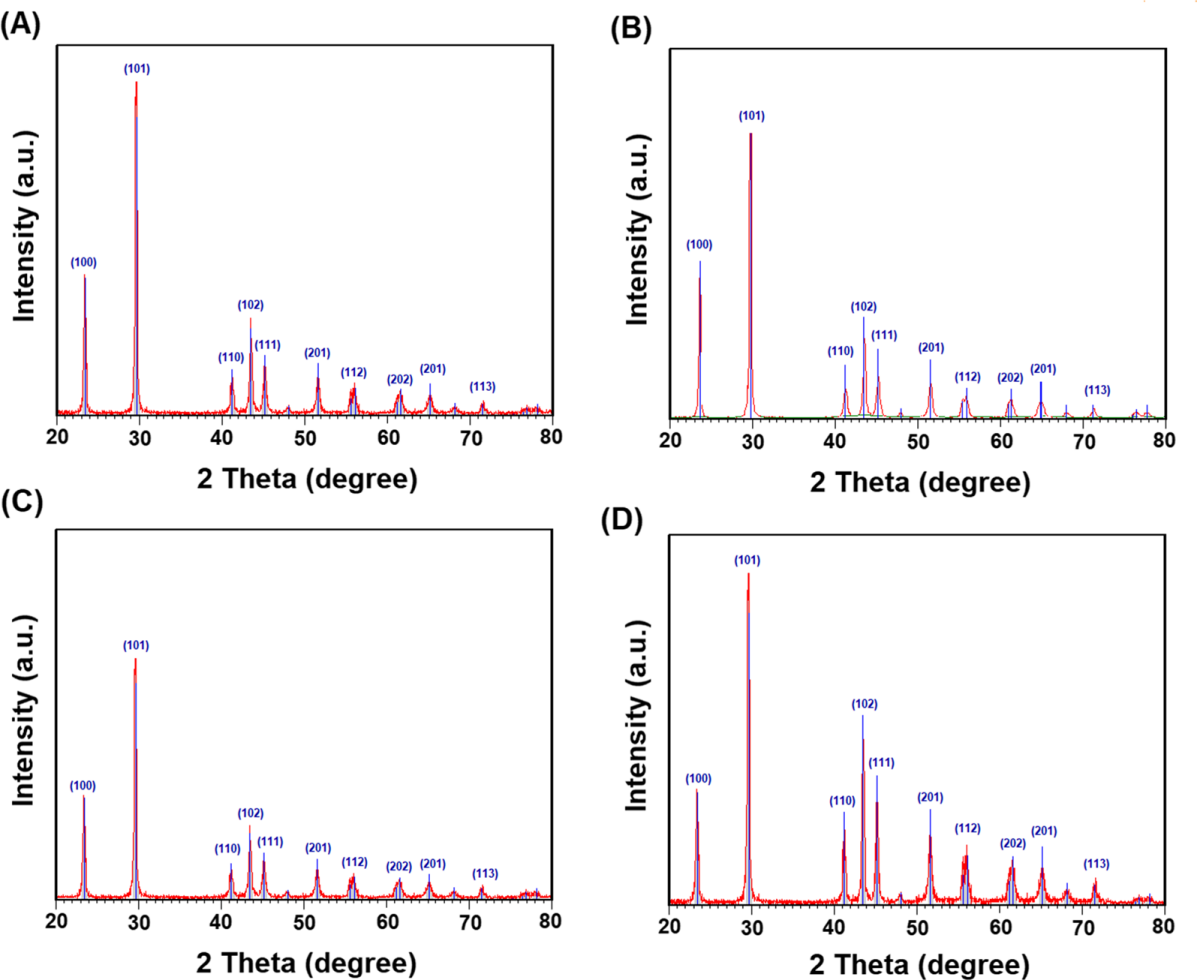
X-ray diffraction patterns (Cu Kα-radiation) of SeNPs at room temperature. **A** XRD pattern of SeNPs synthesized by *A. quadrilineatus*, **B** XRD pattern of SeNPs synthesized by *A. ochraceus*, **C** XRD pattern of SeNPs synthesized by *A. terreus*, and **D** XRD pattern of SeNPs synthesized by *F. equiseti*

Crystal structure and crystallite size of SeNPs synthesized by the four fungal strains were studied by XRD analysis. Figure 3 presented the XRD patterns of SeNPs synthesized by the four fungal strains. The presence of the following planes 100, 101, 110, 102, 111, 201, 112, 202, 201, and 113 in the recorded patterns confirmed the crystal structure of the four types of SeNPs (Fig. 3 A for *A. quadrilineatus*, B for *A. ochraceus*, C for *A. terreus*, and D for *F. equiseti*). Additionally, the crystallite sizes of the four types of SeNPs were estimated from the FWHM of the most intense peak using the Scherrer equation. Data presented in Table 2 showed that the calculated sizes were 55.37 nm for SeNPs synthesized by *A. quadrilineatus*, 45.22 nm for SeNPs synthesized by *A. ochraceus*, 30.98 nm for SeNPs synthesized by *A. terreus*, and 30.11 nm for SeNPs synthesized by *F. equiseti*.

**Table 2:** Calculated size, mean size, size distribution, zeta potential values and polydispersity index (PDI) of SeNPs synthesized by *Aspergillus quadrilineatus*, *Aspergillus ochraceus*, *Aspergillus terreus*, and *Fusarium equiseti*

Studied parameter	Fungal strain			
	*A. quadrilineatus*	*A. ochraceus*	*A. terreus*	*F. equiseti*
Calculated size (nm)	55.37	45.22	67.63	75.13
Size distribution (nm)	20–60	25–75	10–80	20–90
Mean size (nm)	53.21	45.05	65.21	72.15
Zeta potential (mV)	− 27.10	− 15.30	− 16.70	− 12.40
PDI	0.181	0.294	0.466	0.786

Particle size was calculated from the Scherrer equation. Mean size, size distribution, zeta potential and PDI were obtained from the DLS analysis as described in Materials and Methods

#### DLS analyse

Particle size range, particle size means, polydispersion index (PDI) and the zeta potential of SeNPs synthesized by the four fungal strains were studied by DLS analyses. The obtained data (Table 2) clearly showed that distribution of the particle sizes of all the four types of SeNPs was in the range 10–90 nm. Moreover, data presented in Table 2 showed that the recorded mean sizes for the DLS analysis were 53.21, 45.05, 65.21, and 72.15 nm for SeNPs synthesized by *A. quadrilineatus*, *A. ochraceus*, *A. terreus*, and *F. equiseti*, respectively. The recorded PDI-values were 0.181, 0.294, 0.466, and 0.786, respectively. Furthermore, zeta potential values of the respective SeNPs were − 27.10, − 15.30, − 16.70, and − 12.40 mV (Table 2). To test their stability over time, all the four types of SeNPs were separately dispersed in aqueous solutions and checked for successive three months. Interestingly, all the synthesized SeNPs showed excellent stability for three months, where no visual changes or precipitations were observed.

#### TEM studies

**Fig. 4:**
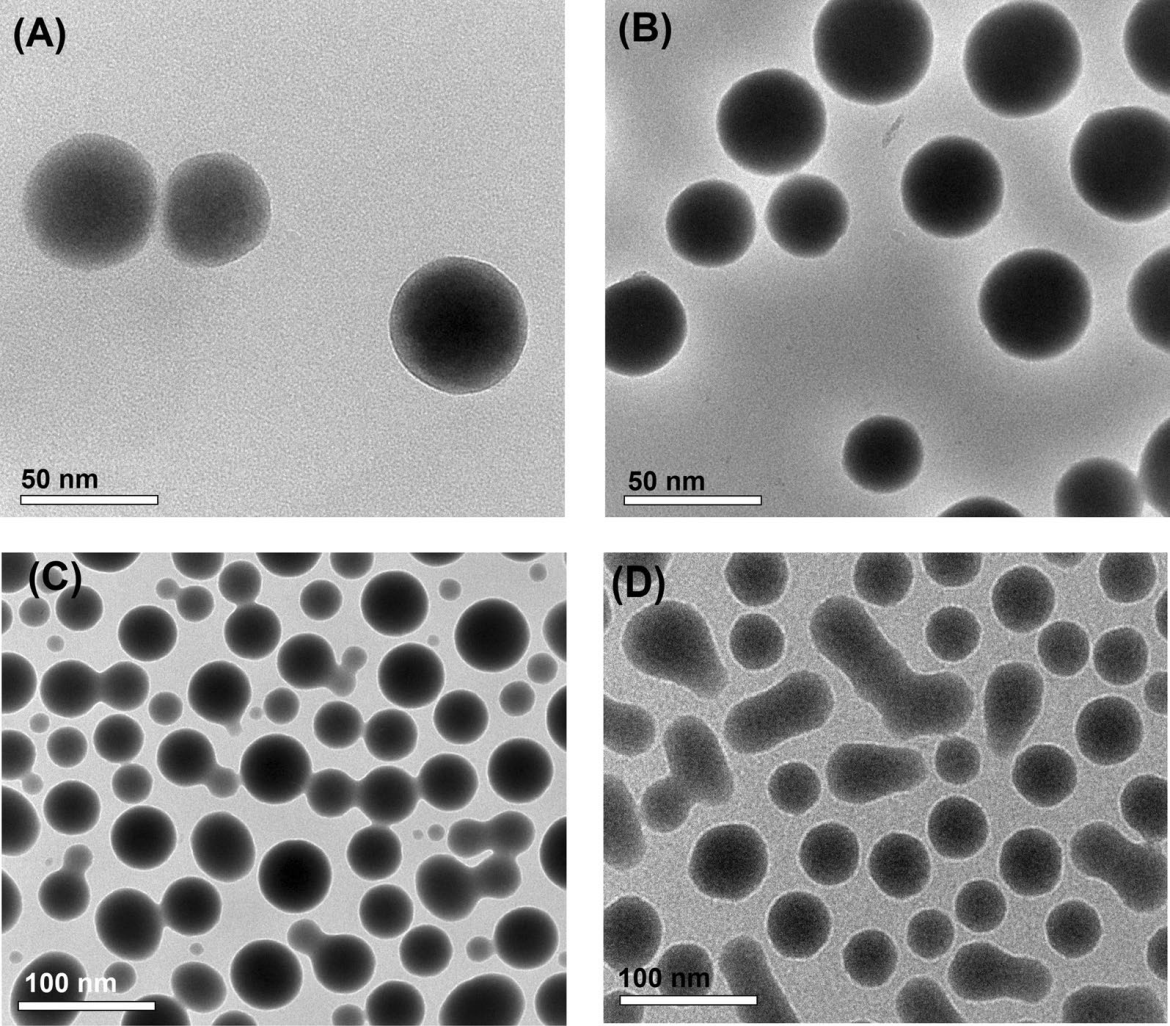
High Resolution TEM analyses of SeNPs. **A** TEM micrograph of SeNPs synthesized by *A. quadrilineatus*, **B** TEM micrograph of SeNPs synthesized by *A. ochraceus*, **C** TEM micrograph of SeNPs synthesized by *A. terreus*, and **D** TEM micrograph of SeNPs synthesized by *F. equiseti*

Morphology of SeNPs synthesized by the four fungal strains were studied by high resolution Transmission electron microscopy (Fig. 4). TEM micrographs of SeNPs synthesized by *A. quadrilineatus* (Fig. 4A) and *A. ochraceus* (Fig. 4B) revealed that the regular spherical shape of SeNPs. The TEM micrograph of SeNPs synthesized by *A. terreus* (Fig. 4C) revealed that the synthesized SeNPs were also spherical in shape but with varying sizes. Moreover, the TEM micrograph of SeNPs synthesized by *F. equiseti* (Fig. 4D) showed that the synthesized NPs were of two shapes, spherical and rod-shaped.

#### FTIR spectroscopy

**Fig. 5:**
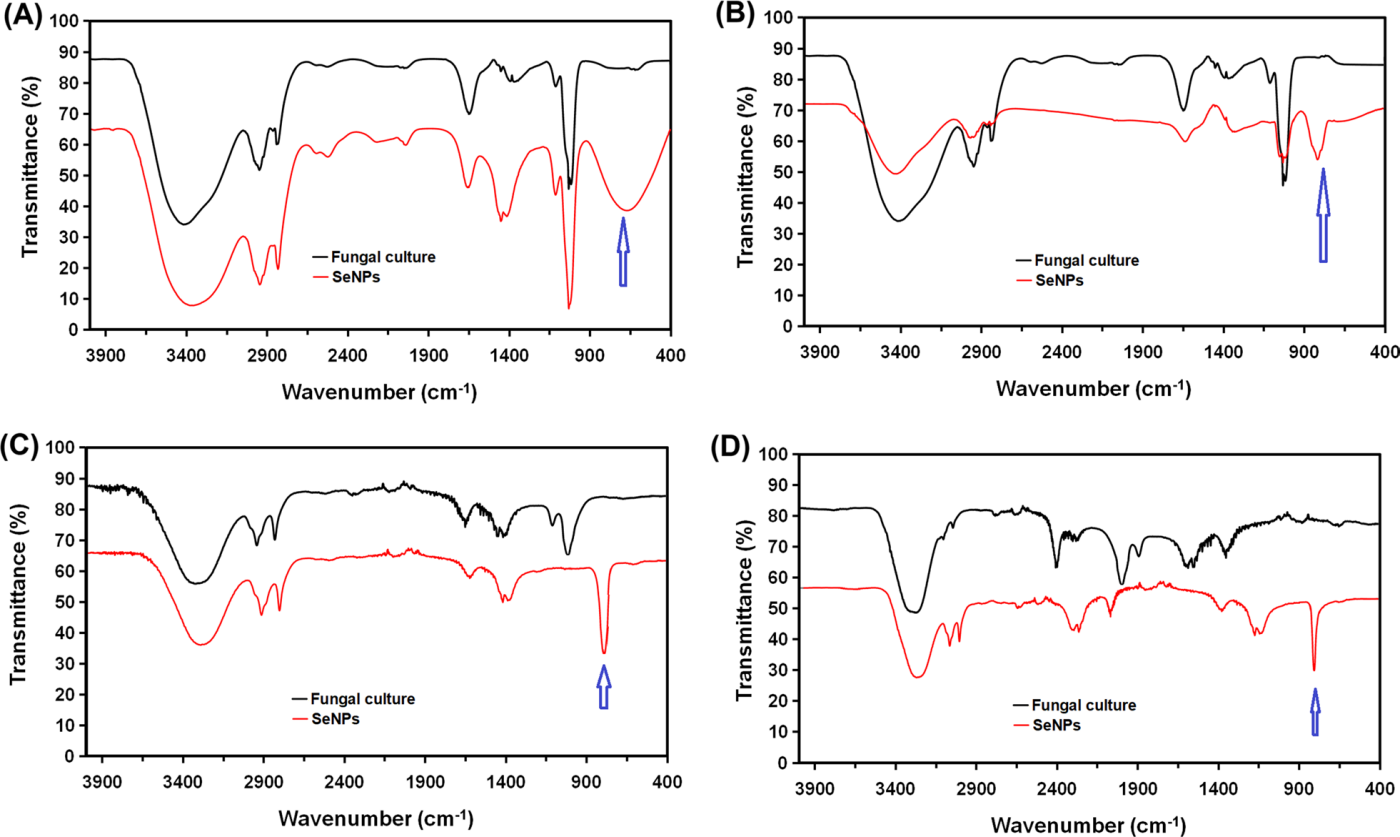
FTIR spectra of the fungal culture (Black line) and the synthesized SeNPs (Red line). **A** FTIR spectrum of SeNPs synthesized by *A. quadrilineatus*, **B** FTIR spectrum of SeNPs synthesized by *A. ochraceus*, **C** FTIR spectrum of SeNPs synthesized by *A. terreus*, and **D** FTIR spectrum of SeNPs synthesized by *F. equiseti*

Formation of SeNPs by the four strains and the interaction between the synthesized SeNPs and active metabolites of the four fungal strains were further investigated by FTIR-spectroscopic analyses. FTIR spectra (Fig. 5) of culture filtrates of the four strains (black line) as well as the four types of SeNPs (red line) were recorded (in the range 400–4000 cm^− 1^). Figure 5 confirmed the apperance of new bands in SeNPs’ spectrum which could be attributed to conjugation. These bands were observed at 621 cm^− 1^ for SeNPs synthesized by *A. quadrilineatus* (Fig. 5 A), 627 cm^− 1^ for SeNPs synthesized by *A. ochraceus* (Fig. 5B), 648 cm^− 1^ for SeNPs synthesized by *A. terreus* (Fig. 5 C) and 652 cm^− 1^ for SeNPs synthesized by *F. equiseti* (Fig. 5D). Nevertheless, in the spectrum of the cultures of the four strains, no bands corresponding bands were observed (Fig. 5). Data further indicated that both spectra of the culture filtrates from the four strains as well as the four types of SeNPs showed bands of phenols, primary amines, C = O and C-O bonds in COO^−^ groups, C-H in CH_2_ and in the phenyl ring, and O-H in water, confirming the impact of active metabolites of the four fungal strains in the synthesis of SeNPs.

### Antifungal activity of SeNPs

The antifungal potential of SeNPs synthesized by the four strains against human and plant pathogenic fungi were investigated by agar-well diffusion assay. Data presented in Table 3 indicated that all SeNPs synthesized by the four fungal strains are promising antifungals when compared by Nystatin. Table 3 also indicated that the MIC-values of SeNPs synthesized by the four strains varies according to the tested fungal pathogen and according to the synthesized SeNPs. Data clearly showed that *C. albicans* was the most sensitive fungal pathogen against all SeNPs and the recorded MIC-values against *C. albicans* were 62.5 µg mL^− 1^ for SeNPs synthesized by *A. quadrilineatus* and *A. ochraceus*. Meanwhile, MIC-values against *C. albicans* for SeNPs synthesized by *A. terreus* and *F. equiseti* were 125 and 250 µg mL^− 1^, respectively (Table 3).

**Table 3:** Antifungal activity of SeNPs synthesized by *A. quadrilineatus*, *A. ochraceus*, *A. terreus*, and *F. equiseti* against different human and plant pathogenic fungi

SeNPs concentration (µg mL^− 1^)		Diameter of inhibition zone (mm)			
		*C. albicans*	*A. brasiliensis*	*A. alternata*	*F. oxysporum*
*A. quadrilineatus*	0.00 (C)	0.00^e^	0.00^e^	0.00^d^	0.00^d^
	31.25	0.00^e^	0.00^e^	0.00^d^	0.00^d^
	62.50	37.67 ± 0.58^d^	0.00^e^	0.00^d^	0.00^d^
	125	44.00 ± 0.58^c^	17.00 ± 1.00^d^	0.00^d^	0.00^d^
	250	45.67 ± 1.00^c^	22.33 ± 2.08^c^	12.67 ± 2.08^c^	15.00 ± 1.00^c^
	500	51.33 ± 2.08^b^	27.00 ± 1.00^b^	19.00 ± 0.58^b^	21.67 ± 0.58^b^
	1000	57.00 ± 1.00^a^	32.67 ± 0.58^a^	25.33 ± 0.58^a^	28.00 ± 2.00^a^
*A. ochraceus*	0.00 (C)	0.00^d^	0.00^d^	0.00^d^	0.00^d^
	31.25	0.00^d^	0.00^d^	0.00^d^	0.00^d^
	62.50	30.67 ± 0.58^c^	0.00^d^	0.00^d^	0.00^d^
	125	41.00 ± 2.00^b^	12.67 ± 0.58^c^	0.00^d^	0.00^d^
	250	43.67 ± 0.58^b^	19.00 ± 1.00^b^	11.67 ± 0.58^c^	11.00 ± 2.00^c^
	500	51.33 ± 2.08^a^	25.33 ± 2.08^a^	17.00 ± 1.00^b^	18.67 ± 0.58^b^
	1000	52.00 ± 0.58^a^	29.00 ± 1.00^a^	20.67 ± 2.08^a^	22.33 ± 2.08^a^
*A. terreus*	0.00 (C)	0.00^d^	0.00^c^	0.00^c^	0.00^c^
	31.25	0.00^d^	0.00^c^	0.00^c^	0.00^c^
	62.50	0.00^d^	0.00^c^	0.00^c^	0.00^c^
	125	0.00^d^	0.00^c^	0.00^c^	0.00^c^
	250	18.58 ± 1.53^c^	0.00^c^	0.00^c^	0.00^c^
	500	24.67 ± 1.00^b^	15.33 ± 2.08^b^	14.67 ± 0.58^b^	15.00 ± 1.00^b^
	1000	31.00 ± 2.00^a^	22.67 ± 1.00^a^	20.00 ± 1.00^a^	21.00 ± 2.00^a^
*F. equiseti*	0.00 (C)	0.00^d^	0.00^c^	0.00^c^	0.00^e^
	31.25	0.00^d^	0.00^c^	0.00^c^	0.00^e^
	62.50	0.00^d^	0.00^e^	0.00^e^	0.00^e^
	125	0.00^d^	0.00^c^	0.00^c^	0.00^e^
	250	16.33 ± 2.08^c^	0.00^c^	0.00^c^	0.00^e^
	500	22.00 ± 1.00^b^	14.33 ± 1.00^b^	16.67 ± 1.00^b^	14.67 ± 0.58^b^
	1000	29.33 ± 2.08^a^	21.58 ± 1.00^a^	22.33 ± 0.57^a^	20.00 ± 1.00^a^
Nystatin/Fluconazole		15.00 ± 1.00	16.33 ± 2.08	13.00 ± 1.00	10.33 ± 0.58

Nystatin/Fluconazole was used at a concentration of 100 µg mL^− 1^. Calculated mean is for triplicate measurements from three independent experiments ± SD, ^a − e^Means with different superscripts in the same column for each fungal strain’s nanoparticle are considered statistically different (LSD test, *P* ≤ 0.05)

### Antibacterial activity of SeNPs

The antibiotic potential of SeNPs synthesized by the four strains were investigated by agar-well diffusion assay against different pathogenic bacteria; Gram-negative and Gram-positive. Table 4 presented the recorded values of the antimicrobial potential of SeNPs synthesized by the four strains against six different human pathogenic bacterial strains. It is very clear from the recorded data that all the synthesized SeNPs are broad spectrum antibiotics when compared with the standard Amoxicillin/Clavulanic acid.

**Table 4:** Antibacterial activity of SeNPs synthesized by *A. quadrilineatus*, *A. ochraceus*, *A. terreus*, and *F. equiseti* against different Gram-positive and Gram-negative human pathogenic bacterial strains

SeNPs concentration (µg mL^− 1^)		Diameter of inhibition zone (mm)					
		*E. coli*	*B. cereus*	*B. subtilis*	*S. aureus*	*P. aeruginosa*	*K. pneumoniae*
*A. quadrilineatus*	0.0 (C)	0.00^e^	0.00^e^	0.00^e^	0.00^d^	0.00^d^	0.00^d^
	31.25	0.00^e^	0.00^e^	0.00^e^	0.00^d^	0.00^d^	0.00^d^
	62.50	18.00 ± 1.00^d^	0.00^e^	0.00^e^	0.00^d^	0.00^d^	0.00^d^
	125	25.67 ± 0.58^c^	12.67 ± 0.58^d^	15.00 ± 1.00^d^	15.33 ± 0.58^c^	0.00^d^	0.00^d^
	250	36.67 ± 1.54^b^	27.00 ± 1.00^c^	24.67 ± 1.54^c^	28.00 ± 1.00^c^	17.67 ± 0.58^c^	17.33 ± 2.08^c^
	500	52.33 ± 2.08^a^	35.67 ± 0.58^b^	33.00 ± 1.00^b^	43.33 ± 1.54^b^	26.00 ± 1.00^b^	27.00 ± 1.00^b^
	1000	55.00 ± 2.00^a^	43.33 ± 2.08^a^	40.00 ± 2.00^a^	54.00 ± 2.00^a^	35.33 ± 1.54^a^	38.00 ± 1.00^a^
*A. ochraceus*	0.0 (C)	0.00^e^	0.00^e^	0.00^e^	0.00^d^	0.00^d^	0.00^d^
	31.25	0.00^e^	0.00^e^	0.00^e^	0.00^d^	0.00^d^	0.00^d^
	62.50	17.33 ± 2.00^d^	0.00^e^	0.00^e^	0.00^e^	0.00^d^	0.00^d^
	125	23.00 ± 1.00^d^	16.33 ± 0.58^d^	11.33 ± 1.54^d^	17.33 ± 0.58^d^	0.00^d^	0.00^d^
	250	34.00 ± 1.00^c^	24.00 ± 1.00^c^	18.00 ± 1.00^c^	26.00 ± 1.00^c^	11.67 ± 2.08^c^	11.00 ± 1.00^c^
	500	42.00 ± 1.00^b^	31.67 ± 2.08^b^	24.33 ± 0.58^b^	32.00 ± 1.00^b^	17.33 ± 0.58^b^	17.33 ± 0.58^b^
	1000	49.67 ± 0.58^a^	39.67 ± 1.54^a^	30.67 ± 2.08^a^	38.67 ± 2.08^a^	24.00 ± 1.00^a^	25.00 ± 2.08^a^
*A. terreus*	0.0 (C)	0.00^d^	0.00^d^	0.00^c^	0.00^c^	0.00^c^	0.00^c^
	31.25	0.00^d^	0.00^d^	0.00^c^	0.00^c^	0.00^c^	0.00^c^
	62.50	0.00^d^	0.00^d^	0.00^c^	0.00^c^	0.00^c^	0.00^c^
	125	0.00^d^	0.00^d^	0.00^c^	0.00^c^	0.00^c^	0.00^c^
	250	14.67 ± 0.68^c^	14.67 ± 2.08^c^	0.00^c^	0.00^c^	0.00^c^	0.00^c^
	500	22.00 ± 1.00^b^	23.00 ± 2.00^b^	17.67 ± 2.08^b^	18.33 ± 1.54^b^	13.33 ± 2.08^b^	14.67 ± 1.54^b^
	1000	31.33 ± 2.08^a^	32.33 ± 1.00^a^	27.00 ± 1.00^a^	35.00 ± 2.00^a^	21.00 ± 1.00^a^	22.00 ± 2.08^a^
*F. equiseti*	0.0 (C)	0.00^e^	0.00^e^	0.00^e^	0.00^e^	0.00^e^	0.00^e^
	31.25	0.00^e^	0.00^e^	0.00^e^	0.00^e^	0.00^e^	0.00^e^
	62.50	0.00^e^	0.00^e^	0.00^e^	0.00^e^	0.00^c^	0.00^e^
	125	0.00^e^	0.00^e^	0.00^e^	0.00^e^	0.00^c^	0.00^c^
	250	14.00 ± 1.00^d^	13.67 ± 0.58^d^	0.00^e^	0.00^e^	0.00^c^	0.00^e^
	500	21.67 ± 1.54^d^	19.67 ± 2.08^d^	16.00 ± 2.00^b^	18.67 ± 1.54^b^	15.00 ± 1.00^b^	12.33 ± 2.08^b^
	1000	30.00 ± 2.00^d^	27.00 ± 1.00^d^	25.00 ± 1.00^a^	26.33 ± 2.08^a^	23.00 ± 1.00^a^	18.33 ± 1.54^a^
Amoxicillin/Clavulanic acid		18.33 ± 2.08	16.67 ± 0.58	17.00 ± 2.00	11.67 ± 1.54	10.00 ± 2.00	13.33 ± 1.54

Amoxicillin/Azithromycin was used at a concentration of 100 µg mL^− 1^. Calculated mean is for triplicate measurements from three independent experiments ± SD, ^a − e^Means with different superscripts in the same column for each fungal strain’s nanoparticle are considered statistically different (LSD test, *P* ≤ 0.05)

Data presented in Table 4 further confirmed that the MIC-values of SeNPs formed by the four strains varied according to SeNPs and the bacterial pathogen. Both the bacterial species *K. pneumoniae* and *P. aeruginosa* were the most resistant pathogens against SeNPs. Despite that, *E. coli* was the most sensitive bacterial pathogen against SeNPs synthesized by the four strains. The obtained data (Table 4) also showed that MIC-values of SeNPs against *E. coli* were 62.50 µg SeNPs mL^− 1^ for either *A. quadrilineatus* or *A. ochraceus*. Meanwhile, MIC-value for SeNPs synthesized by *A. terreus* and *F. equiseti* was 250 µg mL^− 1^.

### Antioxidant behavior of SeNPs

The antioxidant behavior of SeNPs synthesized by the four fungal strains was investigated by the DPPH free radical scavenging assay Data presented in Table 5 showed that SeNPs synthesized by the four fungal strains are promising antioxidants when compared to the standard antioxidant ascorbic acid. From the recorded data, the least inhibitory concentration of both the synthesized SeNPs and ascorbic acid was at a concentration of 25 µg SeNPs mL^− 1^. Furthermore, a dose-dependent manner of SeNPs synthesized by the four fungal strains in inhibition of the DPPH was observed, as the increase in SeNPs concentration significantly increased the scavenging activity (Table 5). Furthermore, the recorded IC_50_-values of SeNPs synthesized by the four fungal strains were 91.52 µg mL^− 1^ for SeNPs synthesized by *A. quadrilineatus*, 100.31 µg mL^− 1^ for SeNPs synthesized by *A. ochraceus*, 151.23 µg mL^− 1^ for SeNPs synthesized by *A. terreus*, and 198.32 µg mL^− 1^ for SeNPs synthesized by *F. equiseti*. Meanwhile, ascorbic acid had an IC_50_ of 70.55 µg mL^− 1^. Accordingly, SeNPs synthesized by the four fungal strains could be arranged according to their antioxidant potential in the descending order as follows: *A. quadrilineatus *>* A. ochraceus *>*A. terreus* >* F. equiseti*.

**Table 5:** DPPH free radical scavenging activity of SeNPs synthesized by *A. quadrilineatus*, *A. ochraceus*, *A. terreus*, and *F. equiseti*

SeNPs concentration (µg mL^− 1^)	Free radical scavenging activity (%)				
	Ascorbic acid	*A. quadrilineatus*	*A. ochraceus*	*A. terreus*	*F. equiseti*
0.00 (C)	00.00 ± 0.00^ g^	00.00 ± 0.00^ h^	00.00 ± 0.00^ h^	00.00 ± 0.00^ g^	00.00 ± 0.00^ h^
25	21.33 ± 1.52^f^	25.76 ± 2.05^ g^	28.38 ± 2.56^ g^	18.00 ± 3.51^f^	10.33 ± 2.06^ g^
50	45.87 ± 1.06^e^	47.33 ± 1.66^f^	46.17 ± 7.33^f^	32.41 ± 1.77^e^	23.86 ± 1.49^f^
100	54.09 ± 5.77^d^	58.78 ± 5.31^e^	50.22 ± 5.42^e^	41.57 ± 7.42^d^	38.54 ± 4.77^e^
200	62.78 ± 4.21^c^	68.06 ± 5.42^d^	58.05 ± 1.78^d^	51.88 ± 5.77^c^	50.01 ± 2.84^d^
400	78.41 ± 6.43^b^	72.67 ± 8.11^c^	67.32 ± 7.21^c^	62.19 ± 2.89^b^	61.28 ± 9.06^c^
800	96.89 ± 8.55^a^	85.45 ± 7.63^b^	75.43 ± 5.73^b^	70.10 ± 5.41^a^	70.51 ± 5.38^b^
1000	100.00 ± 0.00^a^	93.76 ± 9.53^a^	83.59 ± 6.28^a^	79.15 ± 9.33^a^	79.84 ± 4.67^a^
IC_50_ (µg mL^− 1^)	70.55	91.52	100.31	151.23	198.32

DPPH scavenging assay was used for measuring the antioxidant activities of the synthesized nanoparticles at 517 nm using DPPH solution under the conditions described in Materials and Methods. Calculated mean is for triplicate measurements from three independent experiments ± SD, ^a − h^Means with different superscripts in the same column are considered statistically different (LSD test, *P* ≤ 0.05)

## Discussion

Synthesis of nanomaterials using microbial cultures has emerged as a promising biotechnological-based manufacturing process that will aid in the development of more innovative and sustainable industrial nano-manufacturing (Grasso et al. 2021). The achievement of a suitable applicative development of microbial synthesis of nanomaterials is a fascinating emerging research area for the prospective sustainable industrial production. Moreover, fungi were recently suggested as the most promising bio-factories for the production of several nanomaterials (Rao et al. 2017) that will surely open the way for several medical, agricultural, and industrial applications (Zaki et al. 2020; El-Sayed 2021). Thus, we aim in the current study to develop a facile one-pot platforms for preparation for SeNPs. As a first step, 75 endophytic fungal strains were isolated from certain plant species. The isolated were first screened for their efficacy to reduce the precursor sodium selenite and synthesize SeNPs. The isolated strains were then cultivated in PDA plates and PD broth (pH 6.0) amended with sodium selenite solution. The screening profile of these endophytes revealed that four fungal strains were able to reduce sodium selenite solution. Morphological examination of the four fungal strains maintained on the CYA agar (grown at 25 °C for 10 days) were identical with the characteristics concerning the identification of *A. quadrilineatus*, *A. ochraceus*, *A. terreus*, and *F. equiseti* (Moubasher 1993). As such, data obtained from the molecular studies of these strains confirmed the high conformity with their closely related fungi.

In this study, SeNPs synthesized by *A. quadrilineatus*, *A. ochraceus*, *A. terreus*, and *F. equiseti* were separated from the culture broth after incubation and purified, then characterized by several techniques. UV-spectrum of SeNPs formed by the four fungal strains had a UV-maximum absorption peaks at 265 nm. This peak could be attributed to effect of resonance of the SeNPs surface plasmon. The same observations were reported by previous studies concerning the synthesis of SeNPs (Asgari-Paskiabi et al. 2019; Hien et al. 2018; Gunti et al. 2019; Anu et al. 2020). Our results showed that no peaks corresponding to the presence of impurities were observed in the patterns of SeNPs synthesized by the four fungal strains confirming their single-phase nature. In agreement with the Joint Committee on Powder Diffraction Standards cards No. 06-0326 (Asgari-Paskiabi et al. 2019; Mosallam et al. 2018; Zhang et al. 2019) the crystal structure of all the synthesized SeNPs is hexagonal (Anu et al. 2020; El-Sayed et al. 2020a). Results of the DLS-analyses of SeNPs synthesized by *A. quadrilineatus*, *A. ochraceus*, *A. terreus*, and *F. equiseti* showed that the recorded mean size from DLS and particle size distribution matched the calculated size estimated by the Scherrer equation. In addition, SeNPs synthesized by the four fungal strains were monodispersed according to the recorded PDI-values of the DLS-analyses. Also, SeNPs synthesized by the four fungal strains were of high stability according to zeta potential-values taken from the DLS-analyses. Results of the High Resolution TEM analyses of SeNPs synthesized by the four fungal strains indicated the spherical shape of SeNPs synthesized by *A. quadrilineatus*, *A. ochraceus*, and *A. terreus* except for *F. equiseti* it was a mixture of both spherical and rod shapes.

FTIR spectra of SeNPs synthesized by the four fungal strains exhibited the following bands C-H in CH_2_ and in the phenyl ring, vibrations (symmetric and asymmetric) of C-O and C = O in COO^−^ groups, main bands of phenols and primary amines, O-H in H_2_O. Similar observations were reported by previous (El-Baz et al. 2015; Koli et al. 2017; El-Sayed et al. 2020a). However, an absorption band appeared in spectrum of the synthesized SeNPs that is not found in the spectra of the four fungal strains. The formation of these new bands confirms the efficacy of the four strains in the bio-reduction of the precursor Na_2_SeO_4_ salt to SeNPs. Besides, the observed increase in the peak intensity in all spectra of SeNPs which could be attributed to binding of SeNPs to functional groups of the culture of fungi. Similarly, the same observations have been interpreted by El-Sayed et al. (2020a) and Mosallam et al. (2018) who reported the formation of a new bands, besides the increase in intensity of SeNPs peaks. Results of FTIR analyses of SeNPs synthesized by the four fungal strains showed characteristic peaks of proteins, which is responsible for stabilization of the formed SeNPs. Proteins can bind to the formed SeNPs (Butler et al. 2012; Arakelova et al. 2014) by several ways (cysteine residues, free amine groups, and electrostatic attraction of negatively charged carboxylate groups). In this regard, proteins can greatly increase both the biomineralization and stabilization of the synthesized SeNPs (Mosallam et al. 2018; Asghari-Paskiabi et al. 2019; El-Sayed et al. 2020a). Accordingly, these findings suggested that synthesis of SeNPs by the four fungal strains takes place in two successive processes; bio-reduction of selenite salt present in the culture medium and capping the synthesized SeNPs thereby stabilized by metabolites produced by the fungal strain.

Up to date, there is a rapid progress in the research work concerning the fungal biosynthesis of SeNPs. In the literature, twelve fungal strains were reported to have the ability to synthesize SeNPs, including *Monascus purpureus* (El-Sayed et al. 2020a), *Aureobasidium pullulans*, *Mortierella humilis*, *Trichoderma harzianum*, *Phoma glomerata* (Liang et al. 2019), *Mariannaea* sp. HJ (Zhang et al. 2019), *Aspergillus oryzae* (Mosallam et al. 2018), *Lentinula edodes* (Vetc.hinkina et al. 2013), *Aspergillus terreus* (Zare et al. 2013), *Alternaria alternate* (Sarkar et al. 2011), *Fusarium* sp. and *Trichoderma reeii* (Gharieb et al. 1995). Certainly, fungi represent the ideal biotechnological platform for the sustainable production of several nanomaterials (Dorcheh and Vahabi 2016). Besides, the developed one-pot biosynthesis method of SeNPs described in this study for the first time may lower cost of the fungal production of nanomaterials.

In the last two decades, the emerging of antibiotic resistant microbes have become the major health apprehension (Komal et al. 2020) where infectious diseases are the primary causes of deaths around the world (Sundararaj et al. 2019). As a result, there is an emerging utmost concern to develop new antimicrobial formulations to mitigate these drug-resistant microbes. In this context, medicines based on NPs are gaining considerable attention to be used as novel antimicrobial agents. Consequently, SeNPs synthesized by the four fungal strains in this study were evaluated against several pathogens to discover their efficacy as potential antimicrobials. Our results showed that all the synthesized SeNPs are broad-spectrum antimicrobials where growth of all the tested pathogens (fungi or bacteria) were inhibited. In accordance with our results, the antimicrobial activity of SeNPs against several bacterial and fungal species was well reported (Mosallam et al. 2018; Srivastava1 and Mukhopadhyay 2013; Alagesan and Venugopal 2019; Shubharani et al. 2019; El-Sayed et al. 2020a). Several NPs were reported to induce leakage of genetic materials, minerals, and proteins of cells by their interaction with cell wall or membrane (El-Sayed et al. 2020a; Komal et al. 2020; Mousa et al. 2021). As such, they can inhibit the microbial growth by the induction of reactive O_2_ species (Dutta et al. 2012; El-Sayed et al. 2020b; Mousa et al. 2021).

In this study, the antioxidant activity of SeNPs synthesized by the four fungal strains were evaluated. The obtained data revealed their promising antioxidant behavior. In the literature, several reports have reported the promising free radicals scavenging ability of several NPs (Abdelhakim et al. 2020; El-Sayed et al. 2020a; Mousa et al. 2021). The antioxidant potential of several NPs was attributed to the neutralization and inhibition of the formation of free radicals of the DPPH by transfer of electrons (Li et al. 2011; Kovacic and Somanathan 2013, and references therein). Moreover, the high surface to volume ratio can greatly increase the antioxidant behavior of NPs (Das et al. 2013). Our results further indicated that the recorded IC_50_ of SeNPs synthesized by the four fungal strains were in the range 70.55–198.32 µg SeNPs mL^− 1^. Similarly, SeNPs synthesized by culture extract of *Monascus purpureus* showed an antioxidant potential (a dose-dependent manner) at 85.92 µg SeNPs mL^− 1^ (El-Sayed et al. 2020a). In addition, SeNPs synthesized by *Withania somnifera* showed mild antioxidant activity in the range of 20–100 mg mL^− 1^ (Alagesan and Venugopal 2019). Meanwhile, Torres et al. (2012) reported the *in vivo* antioxidant potential of SeNPs synthesized by *P. agglomerans*. Interestingly, the recorded antioxidant potentials of SeNPs synthesized by the four fungal strains in this study are promising in terms of the concentration applied which provides a lead in the exploration of SeNPs as a new source of antioxidants.

In summary, four different endophytic fungal strains were successfully used in a facile-preparation technique of SeNPs. These strains were identified as *A. quadrilineatus, A. ochraceus, A. terreus, and F. equiseti*, based on morphological and molecular studies. SeNPs synthesized by the four fungal strains were characterized by various techniques. The obtained results confirmed their promising action as antifungals, antibacterial, and antioxidants. These findings encouraged us to explore the efficacy of SeNPs in several fields including medicine, food, and agriculture. As a first step, we are currently working on scaling up the process of SeNPs biosynthesis by these promising fungal strains.

## Data Availability

All data generated or analyzed during this study are included in this published article.
